# Perspectives of farmers and veterinarians concerning dairy cattle welfare

**DOI:** 10.1093/af/vfx006

**Published:** 2018-04-11

**Authors:** Christine L Sumner, Marina A G von Keyserlingk, Daniel M Weary

**Affiliations:** Animal Welfare Program, Faculty of Land and Food Systems, University of British Columbia, Vancouver, British Columbia

**Keywords:** disease, pain management, natural living, attitudes

**Implications**

- Dairy farmers and veterinarians share concerns about disease and pain management; however, differences in beliefs about disease prevalence and the pain associated with different conditions and procedures can be barriers to improving animal welfare.
- Misunderstanding of how farmers prioritize animal welfare improvements from both an economic and goal-setting perspective creates a barrier to improvements.
- Farmer and veterinarian perspectives on the natural living construct of animal welfare are not well understood, and future studies are needed to determine how this aspect of welfare can be better addressed.
- Dairy farmer and veterinarian cooperation can improve animal welfare by identifying shared concerns about this issue, reframing unique perspectives as complementary roles, and improving communication about economic priorities and farm goals.

## Introduction

Increasing public concern with the welfare of farmed animals is well documented (Clark et al., 2016). This increased concern by the public, in addition to concerns from within the industry for how farm animals are cared for, has placed pressure on farmers to adopt practices that promote improved welfare (de Rooij et al., 2010). A challenge exists with how these stakeholders perceive each other’s concerns. The public believes that farmers and veterinarians are responsible for ensuring welfare on farms (reviewed by Clark et al., 2016). However, the public also considers farmers to be too oriented toward production (Boogaard et al. 2006). In contrast, dairy farmers (de Rooij et al., 2010) and veterinarians (Ventura et al., 2016) often consider public concerns to reflect an ignorance of modern farming.

Considering that farmers (and veterinarians) have a direct role in affecting animal welfare, it is troubling that their voice “has remained rather mute in public discussions” (Driessen, 2012, p. 165). In our view, any discussion of public attitudes needs to include the farmers and veterinarians who are ultimately responsible for deciding and implementing welfare friendly practices on their farms. We therefore focus this review on dairy farmer and veterinarian perspectives on animal welfare.

Animal welfare can be thought of as three overlapping areas of concern that include an animal’s affective state (i.e., how they are feeling), biological functioning (including health), and natural living (e.g., the extent to which the animal uses behavioral adaptions; Fraser et al., 1997). Although some suggest that dairy farmer perspectives about animal welfare are primarily production-oriented (Bruijnis et al., 2013), many farmers also hold a broader view of animal welfare and place value on how the cows feel (Hansson and Lagerkvist, 2016). Similarly, some reports indicate that veterinarians are concerned about a range of issues beyond disease and pain, including calf care, comfortable housing, and the ability to engage in natural behavior (Ventura et al., 2015).

For the purposes of this review, we have focused on concerns related to biological functioning (such as disease management) and concerns related to affective states (such as pain management), because the perspectives of farmers and veterinarians are best documented for these two aspects of welfare. We turn to concerns around natural living at the end of this review and the implications these have on the public’s acceptance of dairy farming.

Dairy farmers believe that veterinarians have an influential role in improving animal welfare (Wolf et al., 2016). In turn, veterinarians believe that farmers are the most important stakeholder for improving welfare (Ventura et al., 2016). These views, and the available evidence that increased dairy farmer–veterinarian cooperation is beneficial for managing disease on farms (Ritter et al., 2015), suggest that cooperation between these two stakeholders is key to promoting cattle welfare. Farmer–veterinarian cooperation can reduce barriers to improvements in dairy cattle welfare by identifying shared concerns about welfare, reframing their unique perspectives as complementary roles, and promoting communication about priorities and goals.

## Identifying Shared Concerns

For the common goal of improving animal welfare, an initial step is to identify where farmers and veterinarians share concerns about welfare issues. Farmer–veterinarian cooperation can establish these shared concerns as common ground from which to promote specific animal welfare improvements.

### Concerns about disease

The perspective of both farmer and veterinarian on how best to manage disease has received considerable attention and provides an area where these stakeholders share many concerns. A recent on-line survey of Canadian dairy stakeholders showed that disease management ranks highly for both farmers and veterinarians (Bauman et al., 2016). Farmer concerns include managing disease related to calf rearing (Boersema et al., 2013), production (Schewe et al., 2015), and biosecurity (O’Hagan et al., 2016). Similarly, veterinarians are concerned about production diseases (Espetvedt et al., 2013), disease treatment (Richens et al., 2016), and biosecurity (Pritchard et al., 2015).

### Concerns about pain management

Farmers and veterinarians typically agree about what types of procedures (Becker et al., 2013), diseases and injuries (Thomsen et al., 2012) are painful. For example, veterinarians and farmers agree that disbudding and dehorning of calves without analgesics is painful (Winder et al., 2016). Interestingly, Thomsen et al. (2012) found that farmers generally rated conditions more painful than veterinarians, but Becker et al. (2013) found the opposite.

Interpreting farmer and veterinarian attitudes toward painful procedures is challenging because it does not necessarily indicate pain relief is provided. Huxley and Whay (2006) found that veterinarians they surveyed on attitudes towards pain thought that surgical removal of calf horns was painful, and nearly all of them provided pain relief during the procedure (local anesthetic); however, only a few provided relief for the pain that persists in the hours after the procedure. Hötzel and Sneddon (2013) reported that of the 15 veterinarian extension agents working in the south of Brazil they interviewed all but one considered dehorning painful, but none recommended the use of pain relief to farmers who dehorned their own calves.

## Identifying Different Perspectives as Complementary

Although dairy farmers and veterinarians share concerns about animal welfare, they also have unique perspectives based on beliefs about when a problem exists and differences in capacities about how to address it.

### Differences in beliefs

Some farmers believe that presence of disease in their herd is an inevitable consequence of farming and thus beyond their control, and often have variable thresholds for when a problem warrants attention (Ritter et al., 2017). Although not as common, differences in thresholds about when a disease should be treated (or not) have also been documented for veterinarians. For example, veterinarian intention to treat mastitis varied in terms of the waiting period following initial diagnosis (Espetvedt et al., 2013).

Desensitization regarding an animal’s response to painful procedures can contribute to lack of pain mitigation by both farmers and veterinarians (Becker et al., 2014). However, other work has reported that exposure to painful procedures increases sensitivity to pain by both farmers and veterinarians (Winder et al., 2016). Although farmers believe disbudding and dehorning to be painful, different thresholds exist for the severity of pain that requires treatment. These different thresholds can be based on the method used to remove the horns and the age of the calf (farmers: Kling-Eveillard et al., 2015; veterinarians: Hötzel and Sneddon, 2013).

Challenges with reducing lameness also stem from differences in thresholds for considering this a problem (Bruijnis et al., 2013) and a lack of consensus among farmers (Leach et al., 2010a). Horseman et al. (2014) argued that the lack of consensus concerning lameness could be linked to the different uses of language to describe symptoms that may under emphasize pain.

### Differences in capacities

A lack of capacity in identifying or reducing animal welfare problems is evident for both farmers and veterinarians. Ritter et al. (2017) provides a discussion of farmer capacity, including lack of awareness about disease. For example, problems such as lameness persist in part because farmers underestimate the problem (Fabian et al., 2014). Failure to properly treat pain may also stem from both farmer and veterinarian lack of awareness of how to assess (Kling-Eveillard et al., 2015), or treat pain (Winder et al., 2016), and understanding the benefits of pain management (Becker et al., 2013). Farmer failure to treat pain may also be due to a lack of knowledge that pain must be treated under certain regulations (Becker et al., 2013).

Some research has indicated that experience in managing disease on farms can mediate the fatalistic view that disease is an inevitable part of dairy farming (Vaarst and Sørensen, 2009). However, based on the persistence of beliefs and lack of capacities that contribute to farmers not recognizing disease, farmer–veterinarian cooperation may provide additional motivation to improve. Jansen et al. (2009) argue that for the issue of lameness, farmers act when they think a problem exists, and that this threshold for determining a problem is different for each farmer. Considering that farmers believe that veterinarians are an influential advisor for many problems (Wolf et al., 2016), the veterinarian is poised to help farmers overcome such barriers. Veterinarian training in disease management and pain relief, coupled with their relationship with their clients, position them to challenge farmer beliefs about what is considered normal and to implement prevention and treatment plans. For issues of pain management, veterinarian involvement in routine procedures may contribute to increased use of pain relief. Winder et al. (2016) found that farms with routine veterinarian visits were more likely to use pain relief during dehorning, and farms who had adopted pain relief often indicated their herd veterinarian was influential.

## Communication About Priorities

Veterinarians may not always understand how their clients prioritize animal welfare improvements, specifically regarding economic concerns and farm goals (Kristensen and Enevoldsen, 2008). Shortall et al. (2016) describe a potential negative outcome of this poor communication as, “vets and farmers may be talking past each other” (p. 29). Improved communication between farmers and veterinarians may reduce such barriers (Kristensen and Jakobsen, 2011b).

### Economic concerns

Farmer–veterinarian cooperation in improving animal welfare is sometimes hampered by a lack of mutual understanding of how to prioritize economic factors that are variable and context driven. A survey of Canadian farmers found that cost of disease was ranked as a top concern for animal welfare (Bauman et al., 2016), providing some evidence that economic concerns associated with poorly managed disease are important to farmers. However, the nature of this concern is likely context specific. For example, Dutch farmers that had experienced breaches in biosecurity were more concerned with economic loss in contrast to farmers without breaches; the latter being more concerned with costs of prevention (Hop et al., 2011). Canadian dairy farmers enrolled in a Johne’s disease prevention program reported that cost was not a major barrier, with some stating that the program would reduce costs (Sorge et al., 2010), but another Canadian study indicated that dairy farmers found cost and time as the primary barriers to enrollment (Ritter et al., 2015).

Farmer willingness to pay for pain relief is another complex issue. One study reported that although farmers were willing to pay for pain relief during dehorning they were unwilling to cover the total cost (Gottardo et al., 2011). Misch et al. (2007) found that some Canadian dairy farmers who did not use a local block during dehorning cited the cost of drugs as a disincentive for use.

Lameness reduction provides another example of differences in how farmers view the cost of treatment. Leach et al. (2010a) reported that British farmers underestimated the economic loss from lameness and that cost of mitigation methods was a barrier to implementation. Reducing financial losses due to lameness was motivating for Dutch farmers, so long as the measures were perceived as cost-effective (Bruijnis et al., 2013). Tremetsberger and Winckler (2015) provide further discussion on how cost influences farmer motivation to address welfare problems.

Veterinarian perspectives about client willingness to pay can affect their willingness to advocate for improvements. For example, the perspective that clients were not willing to pay for biosecurity was reported as impeding the willingness of veterinarians to approach farmers about this topic (Shortall et al., 2016). Richens et al. (2016) found that veterinarians thought vaccination was an important part of preventing disease on farms, but that willingness to advise use was influenced by their perception of the farmer’s ability to see the economic value in this approach.

Farmers and veterinarians may place different priorities on the cost of mitigating pain. Some studies have found that veterinarians are more concerned than farmers about the cost of pain relief for dehorning (Winder et al., 2016) and the treatment of hoof disorders (Becker et al., 2013). Additionally, there is some evidence that veterinarians overestimate the priority of economic factors as a motivation for farmers. For example, job satisfaction and farm efficiency may be more important to farmers than economic outcomes as motivations to reduce disease (Valeeva et al., 2007). Sorge et al. (2010) found that farmers view the cost of biosecurity measures as less of a concern than the perceived value of the program. Leach et al. (2010b) reported that farmers thought the cost of treatment was the least important barrier to treating lameness; the most motivating reason to reduce lameness was cow pain and suffering.

### Misunderstanding of goals

Veterinarians are trusted advisors for farmers particularly in reference to disease management (Broughan et al., 2016). For example, Leach et al. (2010a) found that British dairy farmers turned more to their herd veterinarian than other sources of information for information regarding lameness reduction. Both stakeholders seem to believe that the veterinarian’s role is to promote health and welfare of the animals (Hall and Wapenaar, 2012), but herd-health programs do not always explicitly target welfare. Fertility and milk production are often the only topics discussed between farmers and veterinarians (Derks et al., 2013a), indicating a missed opportunity to cooperate on issues that more directly address welfare.

A barrier to improving animal welfare through a herd level approach is evident in farmer perspectives on the value of these programs. Some farmers have expressed mixed feelings about the benefits of adopting herd-health programs. Bell et al. (2006) found that although most farmers in their study considered problems such as mastitis and lameness important, 48% did not think herd-health plans would be beneficial in addressing them. Additionally, challenges exist with farmer compliance with herd level programs. For example, Kristensen and Jakobsen (2011a) found that none of the Danish farmers they interviewed had adopted government required biosecurity plans.

Veterinarian services offered through herd-health programs often fail to fully integrate farmer perspectives on improving animal welfare. Kristensen and Enevoldsen (2008) found that farmers placed a higher priority on animal welfare than veterinarians thought they did. Derks et al. (2012) found that only half of the farmers surveyed thought their veterinarian was aware of their farm goals, and almost a quarter felt that they were ignored. In a study on farmer–veterinarian communication about setting goals during herd health, a primary reason for failure to set goals was that veterinarians thought they knew what their clients wanted and that the goal-setting process was too formal (Derks et al. 2013b). Additionally, the reasons farmers gave for not complying with veterinarian advice were related to poor alignment between the advice given and the farm’s goals (Derks et al., 2012). Veterinarians have also admitted that they were often overly critical of farmers, citing lack of education in animal welfare, and poor understanding of the economic barriers facing farmers as barriers to maintaining relationships with their clients (Ventura et al., 2016). Some studies have also found that farmers are interested in advice about disease management and biosecurity (O’Hagan et al., 2016), but veterinarians often assume that farmers are not interested in disease management (Shortall et al., 2016), have limited time for this topic (Richens et al., 2016), have a high tolerance for disease on their farms and fail to place a high priority on biosecurity (Shortall et al., 2016).

Cooperation between farmers and their veterinarians, toward the common goal of improved welfare on farms, will require that veterinarians better understand how farmers prioritize improvements within the context of farm management. Improved communication between these stakeholders may lead to welfare improvements because there is a greater awareness of what each stakeholder values.

## Promoting Cooperation

Examples of farmers and veterinarians partnering to improve farm practices indicate that cooperation can influence the success of such efforts. Examples in the literature indicate that animal welfare improvements based on this cooperation will not be a quick fix; cooperation will need to be an on-going process to sustain welfare improvements. Bell et al. (2009) found that a lack of famer and veterinary compliance with action plans to reduce lameness risks was a reason why an intervention designed to reduce lameness failed. Participating farmers and veterinarians shared concern about lameness reduction, but Bell et al. (2009) found that the participating farmers and veterinarians lacked commitment to implementing action plans. This example suggests that although welfare problems and the root causes can be identified, addressing these can be undermined by poor farmer–veterinarian cooperation.

A study on reducing mastitis on Dutch dairy farms provides an example of where farmer–veterinarian cooperation helped promote adoption of practices that reduce mastitis (Jansen et al., 2010). Participating farmers and veterinarians shared concerns about mastitis, and veterinarians helped develop communication strategies including on-farm study groups targeting topics such as mastitis assessment, goal setting, milking techniques, and an indirect promotion campaign advocating for the use of gloves during milking. The program resulted in increased knowledge and increased interest in controlling mastitis and increased compliance with desired behaviors (using gloves). These authors argued that behavior changes regarding complex issues like mastitis require long-term monitoring to have an impact, and that changing attitudes is an initial step in this direction (Jansen et al., 2010).

## Future Directions

Little is known about farmer–veterinarian perspectives around the natural living aspect of welfare; research is required to understand these concerns and how these relate to those of the general public. Public concerns about welfare are often related to natural living (as reviewed by Clark et al., 2016). A few studies have found that farmers and veterinarians sometimes raise concerns about natural living related to restricted movement due to the use of tie-stalls (Ventura et al., 2015), reduced pasture access in total confinement housing (Schewe and Stewart, 2013), and keeping the cow and calf together after birth (veterinarians: Ellingsen et al., 2012; farmers: Vetouli et al., 2012). Farmers and veterinarians can sometimes dismiss public concerns as based in ignorance. Little is known about how farmers and veterinarians can work together to better understand and address public concerns; however, some evidence exists suggesting dairy farming is adapting to growing public concerns with animal welfare (de Rooij et al., 2010). de Rooij et al. (2010) point to a future where farmer discourses about animal welfare embrace societal concerns.

Promoting naturalness through the reduction of antimicrobials in organic herds is an area where some evidence exists about farmer and veterinarian perspectives. Farmers with organic herds often believe that disease will resolve without conventional treatment (Langford et al., 2009), but veterinarians are often more confident in conventional treatments (Duval et al., 2016). A challenge in improving farmer–veterinarian cooperation in organic dairy farming is shifting the veterinarian’s role from treatment to prevention (Duval et al., 2016). Farmers acknowledge that a lack of dialogue with veterinarians hinders such cooperation (Duval et al., 2017), and it remains to be seen if the farmers and veterinarians can build relationships around antimicrobial usage that meet organic goals and the welfare needs of cattle.

## Conclusion

Improved dairy farmer–veterinarian cooperation may help mediate animal welfare problems. Increased communication between farmers and veterinarians is needed to address respective priorities. Dairy farmers and veterinarians can differ in their perspectives on animal welfare, but also share concerns providing common ground to move forward. Common ground includes improving health, minimizing pain, and to some extent promoting health in organic herds where the focus on naturalness is linked with animal welfare. Improving welfare on dairy farms enables farmers and veterinarians to provide better lives for farm animals and contributes to addressing concerns of the public, which for the foreseeable future, will demand improvements in how farm animals are raised.

## About the Authors

**Dan Weary** is a Professor and NSERC Industrial Research Chair at The University of British Columbia (UBC), Canada. Originally from Quebec, Dan moved to the University of British Columbia in 1997 to co-found the University’s Animal Welfare Program. Dan now co-directs this active group working on a diverse set of research problems.

**Marina (Nina) von Keyserlingk** is a Professor and NSERC Industrial Research Chair at The University of British Columbia (UBC), Canada. After growing up on a beef cattle ranch in British Columbia and completing her Ph.D. at the University of British Columbia, she worked in agri-business for 7 years before joining the UBC Animal Welfare Program in 2002. Together with her colleagues she co-directs a vibrant team made up of undergraduates, graduates, and post-doctoral fellows all devoted to the improving the lives of animals through research, teaching, and outreach.

**Christine Sumner** is currently completing her Ph.D. in the Animal Welfare Program at the University of British Columbia (UBC). Her research focuses on how dairy farmers and veterinarians perceive animal welfare issues and on their motivation to make changes to improve conditions for the animals. She has a keen interest in teaching and is currently working on her certificate in advanced teaching and learning at the post-secondary level. She has worked with exotic and domestic animals in zoological, sanctuary, educational, and farm settings. In addition, she has worked internationally with smallholder livestock farmers and rural development.
